# Artificial Intelligence‐Guided Assessment of Femoral Neck Fractures in Radiographs: A Systematic Review and Multilevel Meta‐Analysis

**DOI:** 10.1111/os.14250

**Published:** 2024-09-27

**Authors:** Nikolai Ramadanov, Jonathan Lettner, Robert Hable, Hassan Tarek Hakam, Robert Prill, Dobromir Dimitrov, Roland Becker, Andreas G. Schreyer, Mikhail Salzmann

**Affiliations:** ^1^ Center of Orthopaedics and Traumatology, Brandenburg Medical School University Hospital Brandenburg an der Havel Brandenburg an der Havel Germany; ^2^ Faculty of Health Science Brandenburg Brandenburg Medical School Theodor Fontane Brandenburg an der Havel Germany; ^3^ Faculty of Applied Computer Science Deggendorf Institute of Technology Deggendorf Germany; ^4^ Department of Surgical Propedeutics, Faculty of Medicine Medical University of Pleven Pleven Bulgaria; ^5^ Institute for Diagnostic and Interventional Radiology, Brandenburg Medical School Theodor Fontane Brandenburg an der Havel Germany

**Keywords:** artificial intelligence, deep learning, femoral neck fractures, hip fractures, meta‐analysis, multilevel meta‐analysis, neural network, radiographs

## Abstract

Artificial Intelligence (AI) is a dynamic area of computer science that is constantly expanding its practical benefits in various fields. The aim of this study was to analyze AI‐guided radiological assessment of femoral neck fractures by performing a systematic review and multilevel meta‐analysis of primary studies. The study protocol was registered in the International Prospective Register of Systematic Reviews (PROSPERO) on May 21, 2024 [CRD42024541055]. The updated Preferred Reporting Items for Systematic Reviews and Meta‐Analysis (PRISMA) guidelines were strictly followed. A systematic literature search of PubMed, Web of Science, Ovid (Med), and Epistemonikos databases was conducted until May 31, 2024. Critical appraisal using the Quality Assessment of Diagnostic Accuracy Studies‐2 (QUADAS‐2) tool showed that the overall quality of the included studies was moderate. In addition, publication bias was presented in funnel plots. A frequentist multilevel meta‐analysis was performed using a random effects model with inverse variance and restricted maximum likelihood heterogeneity estimator with Hartung‐Knapp adjustment. The accuracy between AI‐based and human assessment of femoral neck fractures, sensitivity and specificity with 95% confidence intervals (CIs) were calculated. Study heterogeneity was assessed using the Higgins test *I* ^2^ (low heterogeneity <25%, moderate heterogeneity: 25%–75%, and high heterogeneity >75%). Finally, 11 studies with a total of 21,163 radiographs were included for meta‐analysis. The results of the study quality assessment using the QUADAS‐2 tool are presented in Table 2. The funnel plots indicated a moderate publication bias. The AI showed excellent accuracy in assessment of femoral neck fractures (Accuracy = 0.91, 95% CI 0.83 to 0.96; *I* ^2^ = 99%; *p* < 0.01). The AI showed good sensitivity in assessment of femoral neck fractures (Sensitivity = 0.87, 95% CI 0.77 to 0.93; *I* ^2^ = 98%; *p* < 0.01). The AI showed excellent specificity in assessment of femoral neck fractures (Specificity = 0.91, 95% CI 0.77 to 0.97; *I* ^2^ = 97%; *p* < 0.01). AI‐guided radiological assessment of femoral neck fractures showed excellent accuracy and specificity as well as good sensitivity. The use of AI as a faster and more reliable assessment tool and as an aid in radiological routine seems justified.

## Graphical abstract

Artificial Intelligence (AI) is a dynamic area of computer science that is constantly expanding its practical benefits in various fields. The aim of this analysis which included 11 primary studies with a total of 21,163 radiographs was to analyze AI‐guided radiological assessment of femoral neck fractures by performing a systematic review and multilevel meta‐analysis of primary studies. The AI showed excellent accuracy in assessment of femoral neck fractures. The AI showed excellent sensitivity in assessment of femoral neck fractures. The AI showed excellent specificity in assessment of femoral neck fractures. AI‐guided radiological assessment of femoral neck fractures showed excellent accuracy and specificity as well as good sensitivity. The use of AI as a faster and more reliable assessment tool and as an aid in radiological routine seems justified.

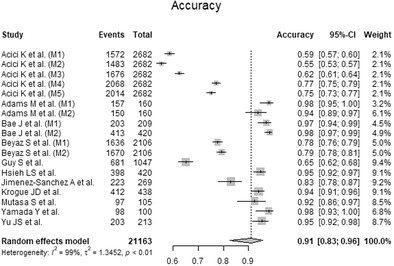

## Introduction

Artificial Intelligence (AI) is a dynamic area of computer science that is constantly expanding its practical benefits in various fields. The overarching goal of AI integration is to provide machines with the intelligence to autonomously perform human tasks. To achieve this, AI systems must have the ability to act adaptively and proactively within their environment. In healthcare, the relentless advance of AI is steadily infiltrating medical applications, with the primary goal of streamlining clinical workflows for physicians. In medicine, concerted efforts are underway to enable AI to accurately diagnose conditions and recommend optimal treatment modalities.^1^ The integration of AI into radiological imaging has become increasingly evident to medical professionals in their daily clinical routine. In orthopedics and traumatology, AI serves as a valuable tool for identifying a spectrum of conditions, including fractures, dislocations, effusions, and bone lesions.^2^ In addition, AI shows a remarkable ability to detect deviations from typical parameters within the human axial skeleton and to assess the progression of osteoarthritis.^3^

AI has demonstrated promising accuracy in diagnosing fractures from radiographs, suggesting promising applications for assisting in the diagnosis of hip radiographs.^2^ Four recently published meta‐analyses^4^, ^5^, ^6^, ^7^ have the major limitation of generalizing to all types of fracture^4^, ^5^ or to all hip fractures^6^, ^7^ without further subdivision into femoral neck fractures, thus failing to take into account the considerable variability between different types of hip fracture. The lack of evidence in the literature highlights the need for this study, which seeks to address this limitation by performing a nuanced analysis specifically targeting femoral neck fractures and their AI‐guided diagnosis. This will help to improve clinical decision‐making and assist healthcare professionals in their decision‐making efforts.^2^

However, before widespread adoption, the efficacy, reliability, and practical utility of AI need to be thoroughly validated in our clinical settings. As a result, there has been a notable increase in the medical literature exploring the role of AI in healthcare.^3^ Femoral neck fractures, being a common injury among the elderly population, underscore the clinical significance of this research endeavor. With an annual incidence of approximately 600–900 per 100,000 persons over the age of 65 in Germany,^8^ there exists a compelling rationale for conducting the first comprehensive multilevel meta‐analysis on this subject.

The aim of this study is to analyze AI‐guided radiological assessment of femoral neck fractures by performing a systematic review and multilevel meta‐analysis of primary studies.

## Methods

The study protocol was registered in the International Prospective Register of Systematic Reviews (PROSPERO) on May 21, 2024 [CRD42024541055]. The updated Preferred Reporting Items for Systematic Reviews and Meta‐Analysis (PRISMA) guidelines^9^ were strictly followed to ensure transparent and comprehensive reporting in line with the principles of scientific rigor. Throughout the process, adherence to the authors' guidelines for meta‐analysis^10^ further enhanced methodological robustness. In accordance with these guidelines, a PRISMA checklist was completed and provided in the supplement.

### Data Sources and Search Strategies

A thorough search of PubMed, Web of Science, Ovid (Med), and Epistemonikos databases using a Boolean search strategy [(((neuronal networks) OR (deep learning) OR (AI) OR (artificial intelligence)) AND ((femoral neck fracture) OR (hip fracture)))] was conducted until May 31, 2024 to ensure a comprehensive collection of relevant literature. Language restrictions were limited to German and English to facilitate inclusivity while ensuring manageable data processing. There were no restrictions on the year of publication.

### Screening and Selection of the Primary Studies

All identified records were transferred to EndNote and duplicates were removed. The abstracts were then screened by two independent reviewers (N.R. and J.L.). Any conflicts were resolved and suitable studies from the abstract screening were included in the full‐text screening. This was also carried out independently by two reviewers (N.R. and J.L.) and any conflicts were also resolved. The final decision on the inclusion or exclusion criteria for each study was made by consensus between the two reviewers (N.R. and J.L.). The necessary information was then extracted from the texts of the included studies.

### Inclusion and Exclusion Criteria

All types of studies with primary data, including randomized controlled trials (RCTs), cohort studies, or case‐control studies in human participants with indications for femoral neck fracture radiographs and AI assessments were regarded as suitable for inclusion. Outcome measures included accuracy, number of radiographs, sensitivity, specificity, and discrimination between fractured and non‐fractured cases in AI‐guided assessment of femoral neck fractures. Case reports, case series, and animal studies were excluded from the analysis.

### Data Extraction

Two reviewers (N.R. and J.L.) independently collected all relevant information regarding study characteristics, methods, quality assessment, participant demographics, accuracy of AI‐guided assessment of femoral neck fractures, and any other relevant supplementary details. The comprehensive dataset extracted from the trials is available in the Supplement. Where accuracy, sensitivity, and specificity values were not explicitly reported in the primary studies, these values were recalculated from other reported numbers whenever possible.

### Quality Assessment of the Primary Studies

Two reviewers (N.R. and J.L.) independently assessed the quality of the primary studies. Critical appraisal was performed using the Quality Assessment of Diagnostic Accuracy Studies‐2 Tool (QUADAS‐2 Tool).^11^ In addition, publication bias was presented in funnel plots.

### Measures of Treatment Effect

A frequentist multilevel meta‐analysis was performed using a random effects model with inverse variance and restricted maximum likelihood heterogeneity estimator with Hartung‐Knapp adjustment. The accuracy between AI‐based and human assessment of femoral neck fractures, sensitivity, and specificity with 95% confidence intervals (CIs) were calculated. Study heterogeneity was assessed using the Higgins test *I* ^2^ (low heterogeneity <25%, moderate heterogeneity: 25–75%, and high heterogeneity >75%).^12^

## Results

### Literature Search

**Figure 1:**
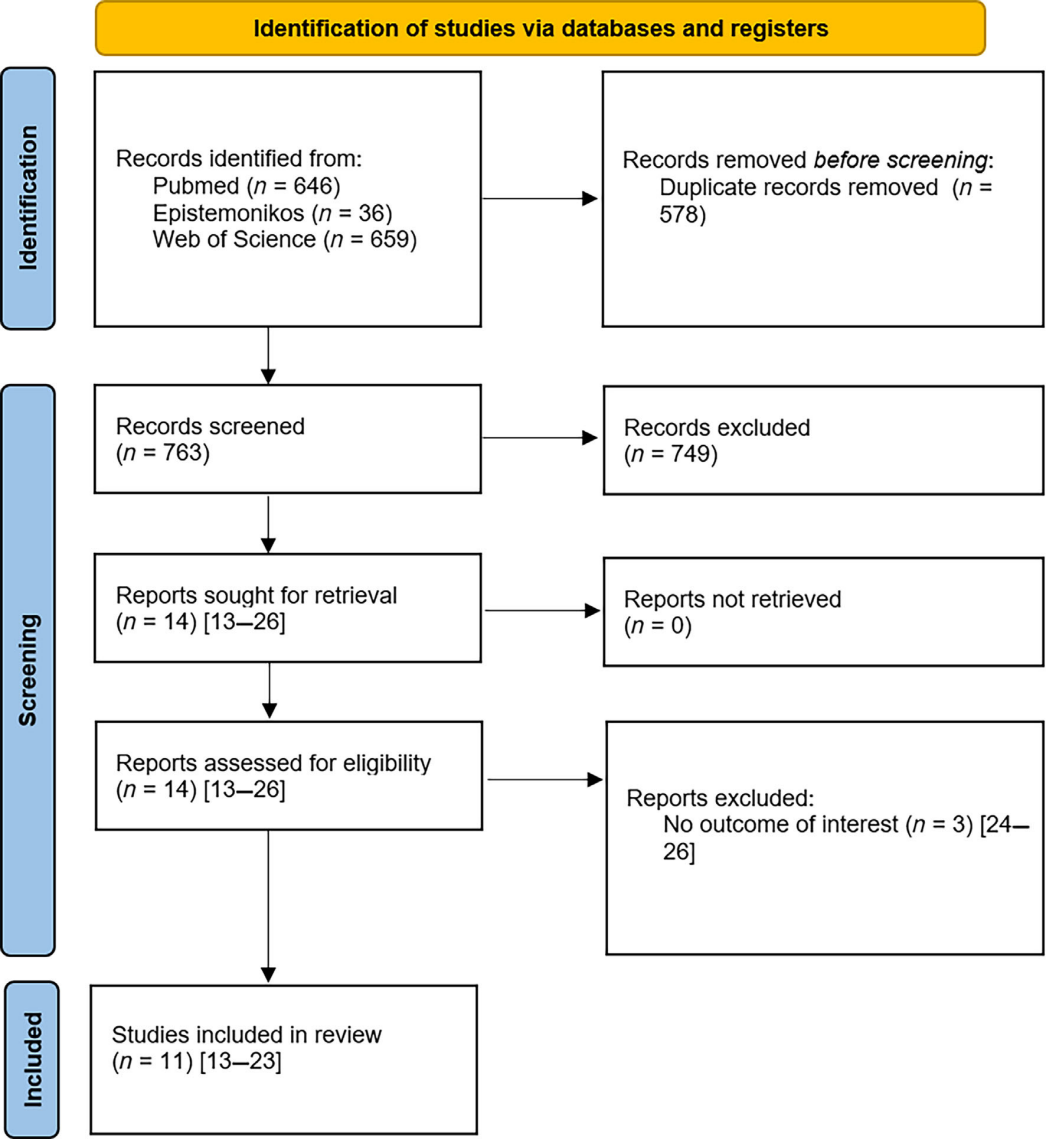
PRISMA flow diagram of the search results and selection according to our inclusion criteria.

After excluding 578 duplicates, the initial literature search yielded 763 records. After title and abstract screening, 14 records remained for further consideration with high inter‐rater agreement (κ = 0.99). Of these 14 studies,^13^, ^14^, ^15^, ^16^, ^17^, ^18^, ^19^, ^20^, ^21^, ^22^, ^23^, ^24^, ^25^, ^26^ 3 were excluded [κ = 1.0] because they did not report the outcome of interest.^24^, ^25^, ^26^ Finally, 11 studies were included for meta‐analysis.^13^, ^14^, ^15^, ^16^, ^17^, ^18^, ^19^, ^20^, ^21^, ^22^, ^23^ The entire search process is shown in a PRISMA flowchart (Figure 1).

### Characteristics of the Studies Included

The 11 included studies were published between 2018 and 2023 and included a total of 21,163 radiographs.^13^, ^14^, ^15^, ^16^, ^17^, ^18^, ^19^, ^20^, ^21^, ^22^, ^23^ Of these radiographs, 10,992 showed a femoral neck fracture and 10,243 showed no fracture. Several studies^13^, ^14^, ^15^, ^16^ reported information on more than one type of AI software for the assessment of femoral neck fractures. The individual types of AI application were calculated separately, as required for a multilevel meta‐analysis. Details of the included studies are shown in Table 1.

**TABLE 1:** Main characteristics of the studies included and the patient cohort.

Author	Year of publication	Origin	Study design	Patients, *N*	Types of radiographs	Radiographs (total), *N*	Fractured (total), *N*
Acici K et al.	2021	Turkey	Experimental study	120	AP pelvis	13,410	6705
Adams M et al.	2018	Australia	Experimental study	NR	AP pelvis, AP hip	320	160
Bae J et al.	2021	South Korea	Retrospective case control study	4189	AP pelvis, AP hip	629	170
Beyaz S et al.	2020	Turkey	Retrospective case control study	65	AP pelvis	4212	2682
Guy S et al.	2021	France	Prospective cohort study	623	AP pelvis, AP hip, lateral hip	1047	391
Hsieh LS et al.	2023	Taiwan	Retrospective cross sectional study	240	AP pelvis	420	420
Jimenez‐Sanchez A et al.	2020	Spain Germany Switzerland France	Experimental study	780	AP pelvis, AP hip	269*	156*
Krogue JD et al.	2020	USA	Retrospective case control study	972	AP pelvis, AP hip, frog‐leg lateral	438	26
Mutasa S et al.	2020	USA	Retrospective case control study	550	AP hip	105	70
Yamada Y et al.	2020	Japan	Retrospective case control study	1262	AP hip, lateral hip	100	50
Yu JS et al.	2019	USA	Retrospective case control study	617	AP pelvis	213	90

Non‐fractured (total), *N*	AI types	Radiographs (multilevel), *N*	Fractured (multilevel), *N*	Non‐fractured (multilevel), *N*	Accuracy	Sensitivity	Specificity
6705	CNN 3C	2682	1341	1341	0.59	0.61	0.56
	CNN with LBP	2682	1341	1341	0.55	0.50	0.60
	CNN with FT	2682	1341	1341	0.63	0.63	0.62
	CNN with GA	2682	1341	1341	0.77	0.76	0.79
	CNN with Particle Swarm Optimization	2682	1341	1341	0.75	0.77	0.73
160	GoogLeNet	160	80	80	0.98	NR	NR
	AlexNet	160	80	80	0.94	NR	NR
459	CNN external	209	59	150	0.97	0.94	0.98
	CNN internal	420	111	309	0.98	0.97	0.99
1530	CNN without GA	2106	1341	765	0.78	0.83	0.69
	CNN with GA	2106	1341	765	0.79	0.83	0.73
656	Tensorflow	1047	391	656	NR	0.61	0.68
0	DAFDNet	420	420	0	0.95	0.95*	NR
113*	automatic CAD tool	269*	156*	113*	0.83*	0.83	0.83
412*	DenseNet	438	26	412*	NR	0.51	0.98
35	CNN	105	70	35	0.92	0.93	0.91
50	CNN	100	50	50	0.98*	1.00	0.98*
123	CNN	213	90	123	0.95*	0.94	0.96

*Note*: AI: Artificial Intelligence; CNN: Convolutional Neural Nnetwork; LBP: local binary patterns; FT: Fourier transformation; GA: genetic algorithm; CAD: computer‐aided diagnosis; *, numbers were calculated; NR: not reported; AP: anteroposterior.

### Quality Assessment

**Figure 2:**
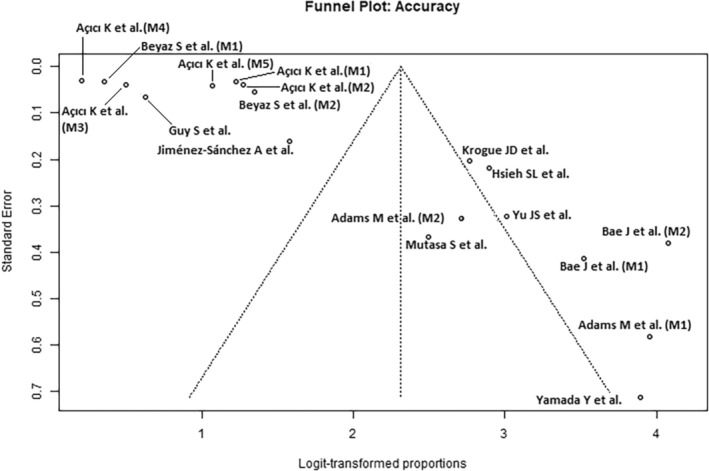
Funnel plots of accuracy for Artificial Intelligence‐guided assessment of femoral neck fractures.

**Figure 3:**
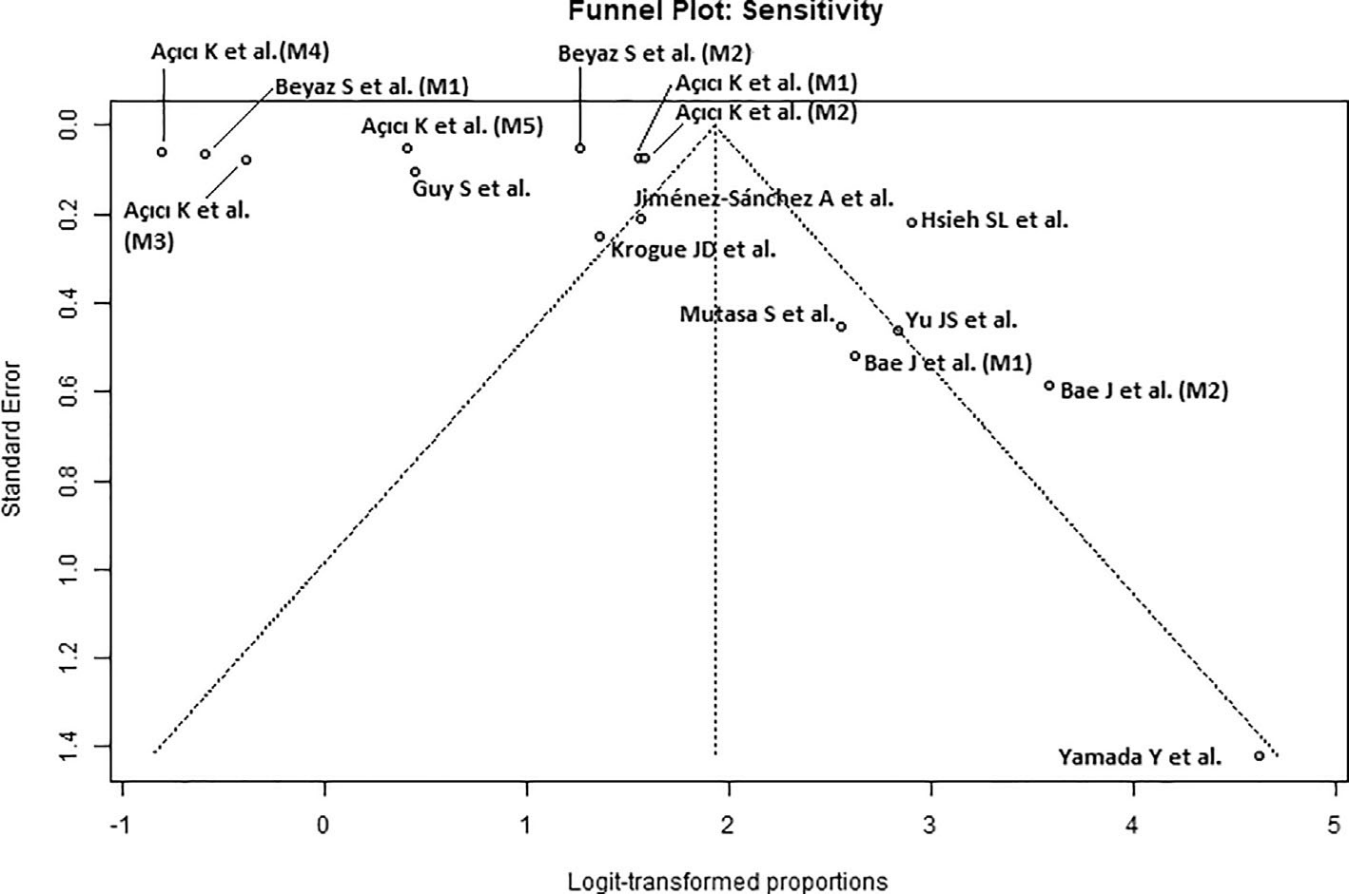
Funnel plots of sensitivity for Artificial Intelligence‐guided assessment of femoral neck fractures.

**Figure 4:**
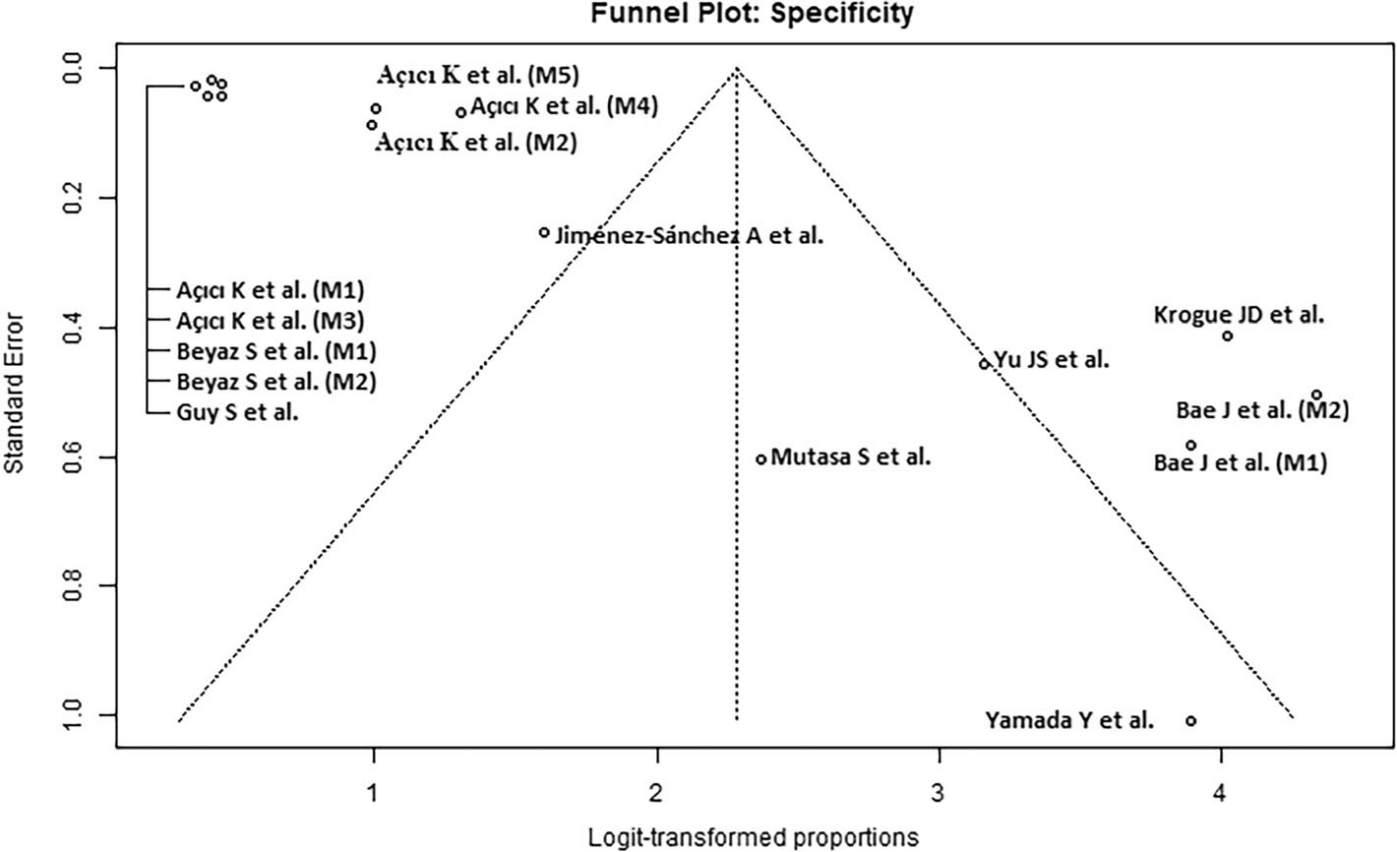
Funnel plots of specifity for Artificial Intelligence‐guided assessment of femoral neck fractures.

The results of the study quality assessment using the QUADAS‐2 Tool are presented in Table 2. The funnel plots (Figures 2, 3, 4) show an uneven distribution of effect sizes across studies around the central line of the overall effect size estimate, indicating moderate publication bias.

**TABLE 2:** Critical appraisal using the Quality Assessment of Diagnostic Accuracy Studies‐2 Tool (QUADAS‐2 Tool).

Study	Q1	Q2	Q3	Q4	Q5	Q6	Q7	Q8	Q9	Q10	% yes	Risk
Açıcı K et al. 2021	U	Y	Y	U	U	Y	U	Y	Y	Y	60%	Moderate
Adams M et al. 2018	U	Y	Y	U	N	Y	U	U	Y	Y	50%	Moderate
Bae J et al. 2021	U	Y	U	U	U	Y	U	U	Y	Y	40%	High
Beyaz S et al. 2020	Y	N/A	U	U	N/A	Y	U	Y	Y	Y	50%	Moderate
Guy S et al. 2021	U	Y	Y	Y	Y	Y	Y	Y	Y	Y	90%	Low
Hsieh SL et al. 2023	Y	N	Y	Y	U	Y	Y	Y	Y	Y	80%	Low
Jiménez‐Sánchez A et al. 2020	U	U	Y	U	U	U	U	U	Y	U	20%	High
Krogue JD et al. 2020	U	Y	Y	Y	N/A	Y	N/A	Y	Y	Y	70%	Moderate
Mutasa S et al. 2020	Y	Y	Y	U	Y	Y	U	Y	Y	Y	80%	Low
Yamada Y et al. 2020	Y	Y	Y	Y	U	Y	Y	U	Y	Y	80%	Low
Yu JS et al. 2019	Y	Y	N	Y	U	Y	Y	Y	Y	N	70%	Moderate

*Note*: Y: yes; N: no; U: unclear; N/A: not applicable; Q1: Was a consecutive or random sample of patients enrolled? Q2: Was a case‐control design avoided? Q3: Did the study avoid inappropriate exclusions? Q4: Were the index test results interpreted without knowledge of the results of the reference standard? Q5: If a threshold was used, was it pre‐specified? Q6: Is the reference standard likely to correctly classify the target condition? Q7: Were the reference standard results interpreted without knowledge of the results of the index test? Q8: Was there an appropriate interval between index test and reference standard? Q9: Did all patients receive the same reference standard? Q10: Were all patients included in the analysis?

## Multilevel Meta‐Analysis

### Accuracy of Artificial Intelligence‐Guided Assessment of Femoral Neck Fractures

**Figure 5:**
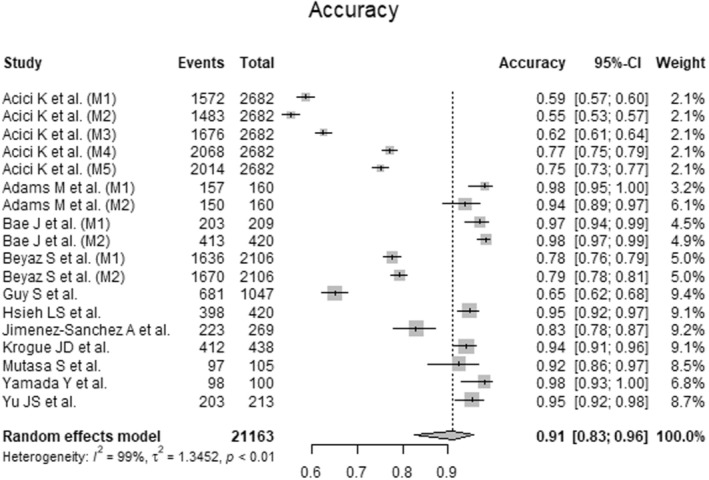
Forest plots of accuracy for Artificial Intelligence‐guided assessment of femoral neck fractures. CI: confidence interval.

To analyze the accuracy of AI‐guided assessment of femoral neck fractures, data from 21,163 radiographs from 11 primary studies were pooled (Figure 5, Table 3). The AI showed excellent accuracy (Accuracy = 0.91, 95% CI 0.83 to 0.96; *I* ^2^ = 99%; *p* < 0.01).

**TABLE 3:** Results of the meta‐analysis for all outcome parameters included.

	Primary studies (multilevel), *N*	Radiographs, *N*	Treatment effect	*I* ^2^	Tau^2^	Egger bias	Egger *p*‐value
Accuracy	18	21,163	0.91	0.99	1,35	N/A	N/A
Sensitivity	16	10,832	0.87	0.98	1,06	N/A	N/A
Specificity	15	10,011	0.91	0.97	2,06	N/A	N/A

*Note*: N/A, not applicable in a multilevel meta‐analysis.

### Sensitivity of Artificial Intelligence‐Guided Assessment of Femoral Neck Fractures

**Figure 6:**
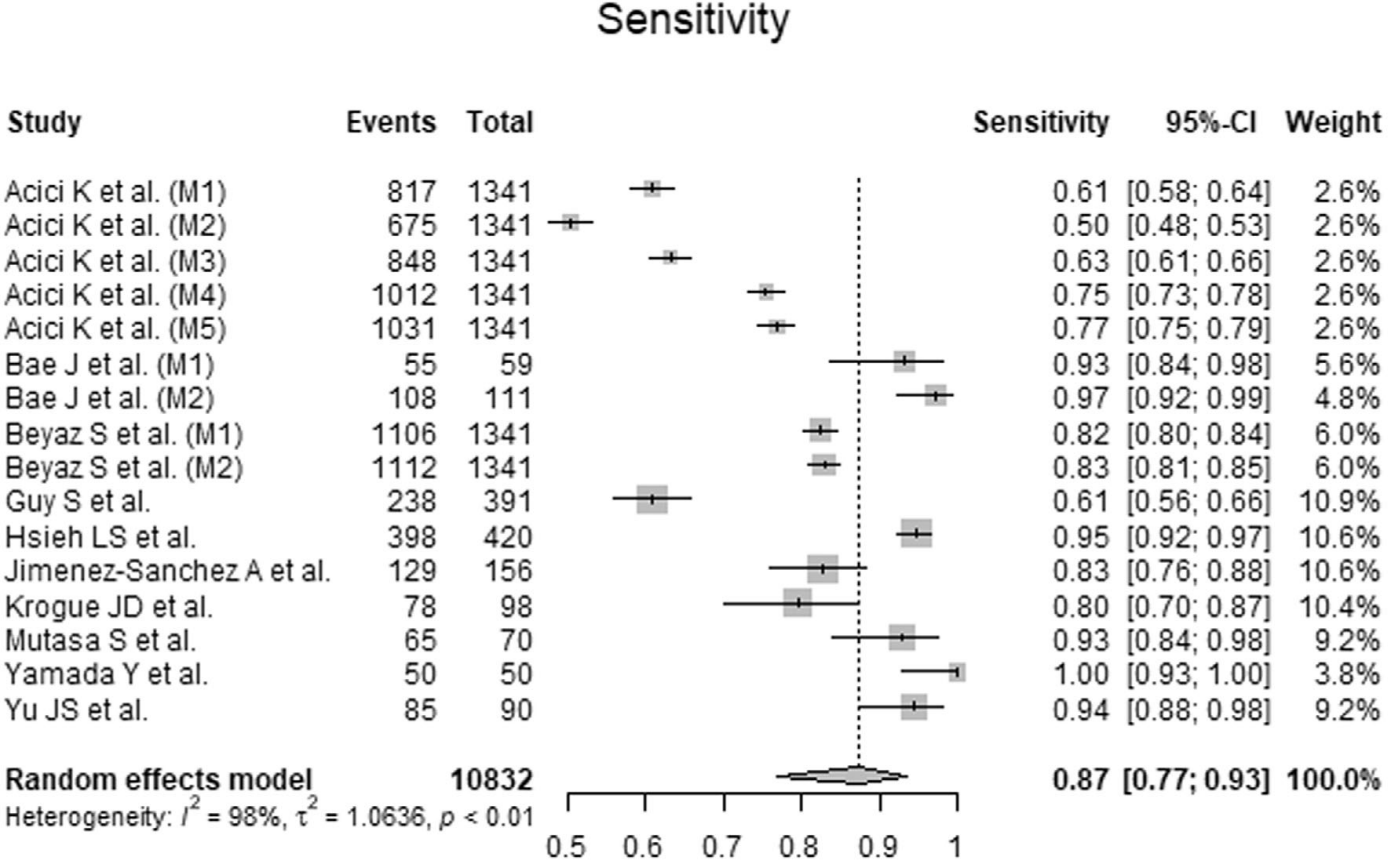
Forest plots of sensitivity for Artificial Intelligence‐guided assessment of femoral neck fractures. CI: confidence interval.

To analyze the sensitivity of AI‐guided assessment of femoral neck fractures, data from 10,832 radiographs from 10 primary studies were pooled (Figure  6 Table 3). The AI showed good sensitivity (Sensitivity = 0.87, 95% CI 0.77 to 0.93; *I* ^2^ = 98%; *p* < 0.01).

### Specificity of Artificial Intelligence‐Guided Assessment of Femoral Neck Fractures

**Figure 7:**
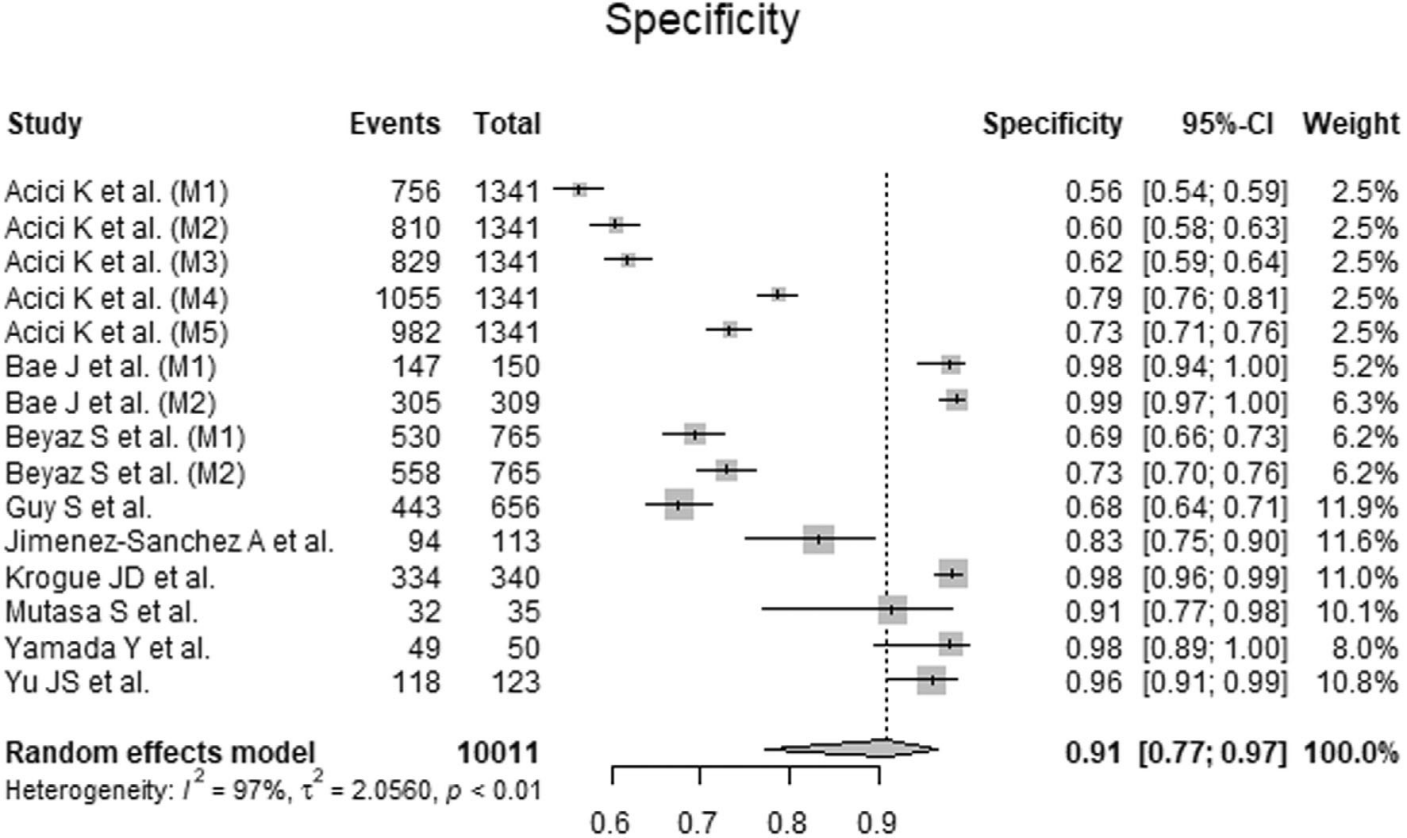
Forest plots of specifity for Artificial Intelligence‐guided assessment of femoral neck fractures. CI: confidence interval.

To analyze the specificity of AI‐guided assessment of femoral neck fractures, data from 10,011 radiographs from nine primary studies were pooled (Figure 7, Table 3). The AI showed excellent specificity (Specificity = 0.91, 95% CI 0.77 to 0.97; *I* ^2^ = 97%; *p* < 0.01).

## Discussion

### Main Findings

The main finding was that AI‐guided radiological assessment of femoral neck fractures showed excellent accuracy of 91%, good sensitivity of 87%, and an excellent specificity of 91%. This means that the AI has comparable abilities to humans and it can be used to reliably assess femoral neck fractures on radiographs in clinical practice. Our multilevel meta‐analysis of AI‐guided radiological assessment of femoral neck fractures showed consistent results with recent similar meta‐analyses on AI fracture detection.^4^, ^5^, ^6^, ^7^

### Implementation in Everyday Clinical Practice

Image processing technology with AI has undergone extraordinary development in recent years. The application of this technology to the analysis of medical images is expected to facilitate the diagnosis of disease and the management of treatment. The use of AI in medical practice could well increase in the coming years, given the rapid pace of technological progress. This raises the question of the implications of this development. AI‐guided fracture assessment seems to be a reliable and very fast partner in everyday radiology.

The promising results of AI in the detection of femoral neck fractures raise the question of its implementation in everyday clinical practice. There, it could be implemented in a variety of ways, depending on the needs of the individual clinical setting. AI tools for femoral neck fracture detection could serve as a new form of automated early warning for radiologists or orthopedic surgeons in cases of femoral neck fracture. This could be useful in hospitals without a full‐time radiologist or in hospitals using teleradiology. In other clinical settings, this tool can be used to triage detected femoral neck fractures to the top of the reading radiologist's queue to speed up the diagnostic process. In other clinical settings, it can simply act as a tool for radiologists to review their decisions. A major advantage is that this automated process works almost instantaneously in real time, which can save considerable time by avoiding delays in human fracture recognition. It can not only improve emergency department efficiency, but also patient outcomes by reducing the time to surgery.

### Displaced and Non‐displaced Femoral Neck Fractures

Unfortunately, few of the primary studies included in the present multilevel meta‐analysis distinguished between non‐displaced (Garden I and II) and displaced (Garden III and IV) femoral neck fractures. Therefore, this distinction could not be taken into account in the present multilevel meta‐analysis. In clinical practice, the diagnosis of a non‐displaced femoral neck fracture is often difficult. Especially when the non‐displaced fracture is submerged (Garden I) and the patient is still able to walk with pain, such fractures are often missed by human assessors. The problem in detecting Garden I femoral neck fractures arises from the difficulty in detecting the disruption or change in orientation of the trabecular trajectories on conventional radiographs. For this reason, computed tomography (CT) or even magnetic resonance imaging (MRI) is often required to reliably diagnose a non‐displaced femoral neck fracture. As we can see, the AI has made great strides in medical imaging. The key question is whether AI can be trained to reliably diagnose non‐displaced femoral neck fractures without the need for further diagnosis using CT or MRI. This would mean that even difficult‐to‐detect femoral neck fractures, namely non‐displaced Garden I and II fractures, could be detected at an early stage. This would significantly improve the outcome of these patients, as the time to surgical treatment is known to be a very important factor in postoperative outcome. Reducing the time to surgery could reduce the length of hospital stay, postoperative complications, and mortality in femoral neck fractures.^27^ The risk of complications increases by 121% and 142% when the time to surgery was 3 days and 4–7 days, respectively.^28^

### Comparison With the Literature

The advantage of the present meta‐analysis over the comparable literature is that it overcomes a major limitation. To the best of our knowledge, this is the first meta‐analysis to examine the performance of AI‐guided radiological assessment of femoral neck fractures. Recently published comparable meta‐analyses^4^, ^5^, ^6^, ^7^ have generalized their study focus to the AI‐guided radiological assessment of all fracture types^4^, ^5^ or to the AI‐guided radiological assessment of hip fractures.^6^, ^7^ The 2022 meta‐analysis of 42 studies by Kuo et al. found a sensitivity of 92% and a specificity of 91% for fractures in general.^4^ The 2022 meta‐analysis of 39 studies by Zhang et al. found an accuracy of 96%, a sensitivity of 90%, and a specificity of 92% for fractures in general.^5^ The 2023 meta‐analysis of 39 studies by Lex et al. found an accuracy of 79%, a sensitivity of 89.3%, and a specificity of 87.5% for hip fractures.^6^ The 2024 meta‐analysis of 66 studies by Jung et al. found that AI in fracture detection had an accuracy of 91%, a sensitivity of 92%, and a specificity of 90% for hip fractures.^7^ Compared with the results of the AI, the sensitivity of the different physician groups in detecting hip fractures is as follows: 69.2 (54.9–81.3) for general practitioners, 73.1 (59.0–84.4) for orthopedic residents, 76.9 (63.2–87.5) for radiology residents, 96.2 (86.8–99.5) for orthopedic chief residents, 92.3 (81.5–97.9) for radiology chief residents, 96.2 (86.8–99.5) for attending orthopedic surgeons, and 96.2 (86.8–99.5) for attending radiologists.^29^ The specificity is 96.6 (92.3–98.9) for general practitioners, 98.0 (94.2–99.6) for orthopedic residents, 98.0 (94.2–99.6) for radiology residents, 97.3 (93.2–99.3) for orthopedic chief residents, 93.9 (88.8–97.2) for radiology chief residents, 97.3 (93.2–99.3) for attending orthopedic surgeons, and 95.3 (90.5–98.1) for attending radiologists.^29^

### Strengths and Limitations

The strengths and limitations of this multilevel meta‐analysis are as follows: (i) As four^13^, ^14^, ^15^, ^16^ of the included primary studies reported the results of multiple types of AI application for radiological assessment of femoral neck fractures, a multilevel meta‐analysis was performed to obtain and synthesize more primary data. (ii) This systematic review and multilevel meta‐analysis of 11 primary studies^13^, ^14^, ^15^, ^16^, ^17^, ^18^, ^19^, ^20^, ^21^, ^22^, ^23^ with 21.163 radiographs is the first attempt to qualitatively and quantitatively review the existing evidence on AI‐guided radiological assessment of femoral neck fractures.

The limitations were as follows: (i) The patient cohorts of the included studies were heterogeneous, combining patients with different types of radiographs. (ii) The AI applications used were also heterogeneous. (iii) There were insufficient primary data to differentiate between displaced and non‐displaced femoral neck fractures. (iv) Some of the numbers in the data extraction from the primary studies had to be calculated and reconstructed. (v) All three outcome measures included in the multilevel meta‐analysis showed a high degree of heterogeneity and a moderate publication bias.

## Conclusion

AI‐guided radiological assessment of femoral neck fractures showed excellent accuracy of 91%, good sensitivity of 87%, and excellent specificity of 91%, demonstrating comparable abilities to humans. Therefore, the use of AI as a faster and more reliable assessment tool and as an aid in radiological routine seems justified.

## Author Contributions

NR and JL performed the search and data extraction. RH and NR performed the statistics. RH, NR, and JL created tables and figures. NR wrote the manuscript. HTH, RP, DD, RB, AGS, and MS performed the correction in the revision process. All authors supervised the whole process and read the final version.

## Conflict of Interest

No conflict of interest.

## Ethics Statement

Not applicable as this is a systematic review.

## Supporting information


**Data S1** Supporting Information.

**Data S2** PRISMA CHECKLIST.

## Data Availability

Raw data extraction sheet is available in Supplement.
